# Antioxidant Activity of Chlorogenic Acid Evaluated via EPR Spectroscopy and Its Visual Tracking in Mouse Kidney

**DOI:** 10.3390/nu18081181

**Published:** 2026-04-09

**Authors:** Li Quan, Cheng Li, Peipei Shen, Enchao Zhou, Gui Yin, Xuewen Guo

**Affiliations:** 1 The First Clinical Medical College, Nanjing University of Chinese Medicine, Nanjing 210023, China; 039317110@njucm.edu.cn; 2 Jiangsu University Key Laboratory of Tonifying Kidney and Anti-Senescence, Nanjing 210023, China; 3 School of Chemistry, Centre for Shared Scientific Research Facilities, Nanjing University, Nanjing 210023, China; licheng@nju.edu.cn (C.L.); spp050300@163.com (P.S.); yingui@nju.edu.cn (G.Y.)

**Keywords:** chlorogenic acid, antioxidant activity, visual tracking, EPR spectroscopy, kidney disease

## Abstract

**Background/Objectives:** Chlorogenic acid (CGA) is a natural antioxidant widely distributed in various plant foods, exhibiting great potential for the development of natural antioxidant agents and biomedical applications. **Methods:** In this study, the antioxidant activity of CGA was first characterized via electron paramagnetic resonance (EPR) spectroscopy by determining its scavenging capacity against 1,1-diphenyl-2-picrylhydrazyl (DPPH) radicals. Meanwhile, its hydroxyl radical (•OH) scavenging activity in aqueous solution was quantitatively evaluated based on the signal intensity changes of DMPO-OH• adducts. Furthermore, a fluorescein-labeled chlorogenic acid derivative (FL-CGA) was utilized to visualize the distribution of CGA in major mouse organs following tail vein injection, with a specific focus on the kidney, and to investigate its penetration capacity into podocytes. **Results:** The results demonstrated that 0.35 mM CGA exerted potent scavenging activity toward highly reactive and cytotoxic •OH radicals, achieving a scavenging rate of 95.2% in a system where •OH was generated by continuous UV irradiation of 5 mM H_2_O_2_ aqueous solution for 30 min. Additionally, FL-CGA was specifically accumulated in the kidney and localized to the lysosomes of podocytes, while no signal was detected in the endoplasmic reticulum or mitochondria. **Conclusions:** This study provides experimental evidence to further elucidate the mechanisms underlying CGA-mediated intervention in renal injury, and lays a foundation for the further development and clinical application of CGA as a natural dietary antioxidant.

## 1. Introduction

Chronic kidney disease (CKD) has emerged as a pressing global public health issue with severe complications and high mortality. Its pathogenesis involves repetitive injury to glomerular/tubular epithelium, accompanied by abnormal cell proliferation/repair, inflammation, fibroblast activation, and fibrosis, ultimately leading to renal dysfunction and end-stage kidney disease [1]. Oxidative stress, which is characterized by elevated intracellular levels of reactive oxygen species (ROS) and highly prevalent in multiple renal diseases [2,3], underpins these pathological processes, playing a key role in renal damage and serving as a potential therapeutic target [4,5,6,7].

Chlorogenic acid (CGA) is a phenolic acid polyphenol formed by the esterification of caffeic acid and quinic acid, which is widely distributed in herbal medicines, coffee, fruits, and vegetables. Extensive research has demonstrated that CGA possesses a range of beneficial biological activities, including potent antioxidant, anti-inflammatory, and immunomodulatory effects, highlighting its considerable potential for the prevention and treatment of renal diseases [8,9,10]. For instance, in a mouse model of renal injury induced by sodium arsenite, CGA was found to alleviate oxidative stress damage, inflammatory infiltration, and cellular apoptosis in renal tissues [11]. In a streptozotocin (STZ)-induced diabetic senescent rat model of renal injury, the combined use of CGA with other polyphenols such as naringin and quercetin exerted a synergistic effect in improving the function of the renal antioxidant defense system and enhancing the protective efficacy [12]. In addition, CGA can also precisely intervene in inflammatory responses and attenuate renal fibrosis through multiple pathways, which represent a core pathological mechanism mediating the occurrence and progression of renal injury. For example, in the treatment of diabetic nephropathy (DN), CGA enhanced antioxidative stress capacity by activating nuclear factor E2-related factor 2 (Nrf2) while inhibiting the activation of the NLRP3 inflammasome to exert anti-inflammatory effects [13]. CGA alleviated renal fibrosis by inhibiting the Notch1 and Stat3 signaling pathways and reducing renal lipid accumulation in treating DN [14].

In the in vitro characterization of CGA antioxidant activity, spectrophotometric methods are conventionally used to assess its scavenging efficacy against typical free radicals, including 2,2′-azino-bis(3-ethylbenzothiazoline-6-sulphonic acid) (ABTS) and 2,2-diphenyl-1-picrylhydrazyl (DPPH) [15,16,17]. As one of the most reactive and cytotoxic members of reactive oxygen species, the hydroxyl radical (•OH) can induce extensive damage to critical cellular components in living organisms [18]. Therefore, evaluating the •OH-scavenging activity of antioxidants is of greater biological relevance; however, studies investigating the direct •OH-scavenging capacity of CGA remain scarce [19].

In addition, fluorescein derivatives can produce intense fluorescence signals upon excitation with ultraviolet or blue light, and have thus been widely utilized in diverse technical fields, including fluorescence microscopy, flow cytometry, immunoassays, and molecular biology-related manipulations [20]. Chemical modification of the fluorescein molecular skeleton enables the introduction of distinct functional groups or substituents, which can further modulate the fluorescence properties of these compounds, such as emission wavelength, fluorescence intensity, and photostability, thereby expanding their applicability across multiple disciplines [21,22,23]. For instance, fluorescein derivatives can be conjugated to antibodies, proteins, or other biomolecules to realize specific targeting and visual tracking of subcellular structures, biomarkers, and intermolecular interactions [24,25,26].

To date, few studies have directly conjugated CGA with fluorescein for real-time tracing of CGA during renal disease intervention. The present study employed a fluorescein-labeled CGA derivative (FL-CGA) as a molecular probe to visualize the therapeutic localization of CGA via fluorescence imaging, aiming to provide direct cellular and subcellular evidence for CGA’s renal protective effects in CKD. Furthermore, we utilized electron paramagnetic resonance (EPR) spectroscopy to quantitatively evaluate the antioxidant activity of CGA, using the signal intensity changes of DMPO-OH• adducts as a reliable indicator to assess its hydroxyl radical (•OH) scavenging capacity in aqueous solution. Collectively, this study comprehensively investigated the in vitro •OH scavenging activity of CGA via EPR and established a FL-CGA-based fluorescence imaging strategy for real-time tracking of CGA’s renal distribution and podocyte uptake. These findings not only fill the current gap in real-time tracing of dietary CGA in renal research but also provide a solid experimental and theoretical basis for further elucidating the molecular mechanisms underlying CGA-mediated antioxidant intervention in renal injury.

## 2. Materials and Methods

### 2.1. Materials

CGA (purity ≥ 98%) was purchased from Shanghai D&B Biological Science and Technology Co., Ltd. (Shanghai, China); DPPH (purity ≥ 98.5%) and fluorescein were obtained from Shanghai Macklin Biochemical Co., Ltd., (Shanghai, China); 5,5-dimethyl-1-pyrroline *N*-oxide (DMPO) was purchased from Dojindo China Co., Ltd. (Shanghai, China). Ethanol and hydrogen peroxide (H_2_O_2_, 30%) were purchased from Nanjing Chemical Reagent Co., Ltd. (Nanjing, China); Dimethyl Sulfoxide (DMSO) and Mycoplasma Elimination Reagent were purchased from Yeasen Biotechnology (Shanghai) Co., Ltd. (Shanghai, China). Fluorescein-labeled chlorogenic acid (FL-CGA) was provided by Chongqing Yusi Pharmaceutical Technology Co., Ltd. (Chongqing, China) (MALDI-TOF MS [FL-CGA+H^+^]: 842.259, found: 842.281).

Cell culture reagents: Fetal bovine serum (FBS) was obtained from Zhejiang Tianhang Biotechnology Co., Ltd. (Huzhou, China). Phosphate-buffered saline (PBS), Roswell Park Memorial Institute-1640 (RPMI-1640) medium, and penicillin-streptomycin double antibody solution were purchased from Shanghai Basalmedia Technology Co., Ltd. (Shanghai, China). The 0.25% trypsin-EDTA solution and cell counting Kit-8 were purchased from Beijing Laijeke Technology Co., Ltd. (Beijing, China). Glucose was purchased from Shanghai Aladdin Biochemical Technology Co., Ltd. (Shanghai, China).

Cell staining reagent: Neutral universal tissue fixative (Cat: G1101-500ML) was purchased from Wuhan Servicebio technology Co., Ltd. (Wuhan, China). 4′,6-diamidino-2-phenylindole (DAPI) was obtained from Beijing Solarbio Science & Technology Co., Ltd. (Beijing, China). The endoplasmic reticulum blue fluorescent probe (ER-Tracker Blue-White DPX) was purchased from Yeasen Biotechnology (Shanghai) Co., Ltd. (Shanghai, China). Mitochondrial red fluorescent probe (MitoRed) and lysosomal red fluorescent probe (Lyso Red DND-99) were purchased from Jiangsu Keygen Biotech Co., Ltd. (Nanjing, China).

### 2.2. Measurement of Antioxidant Activity of CGA by EPR Spectroscopy

All EPR measurements were conducted in accordance with our previous method [27] with some modification as described in Supplementary Materials.

#### 2.2.1. Antioxidant Activity of CGA Against DPPH Radicals

Time-dependence of DPPH radical scavenging activity by CGA: In a 90% ethanol-aqueous solution, an EPR assay was performed on the reaction system containing 0.5 mM DPPH and 0.2 mM CGA, with EPR spectra recorded periodically at different time intervals. The area corresponding to the characteristic DPPH signal peaks was quantified through double integration of the corrected EPR spectra, using Bruker ESR Studio software (Version 1.90.0, Bruker BioSpin GmbH, Ettlingen, Germany). The DPPH radical scavenging rate (SR) is calculated by the following formula, as detailed in Supplementary Material Text S2.

SR = (A_0_ − A)/A_0_ × 100% (1)

where A_0_ represents the double integral area of the characteristic peaks in the EPR spectrum for the blank group, and A corresponds to of the EPR spectra for the sample groups.

Correlation between CGA dosage and DPPH radical-scavenging efficacy: 100 μL of CGA aqueous solutions with an initial concentration range of 0.5–3.5 mM (deionized water served as blank control) was mixed with an equal volume (100 μL) of 5 mM DPPH ethanol solution; subsequently, ethanol was supplemented to adjust the total volume of the mixture to 1000 μL. The resulting reaction system was subjected to dark incubation for 15 min, followed by electron EPR spectroscopic analysis.

#### 2.2.2. Antioxidant Activity of CGA Against •OH

Correlation between CGA dosage and •OH radical-scavenging efficacy: A 50 μL aqueous mixture containing 1 mM DMPO, 5 mM H_2_O_2_, and CGA at a concentration gradient of 0.15–0.45 mM was loaded into glass capillaries and irradiated with a xenon lamp positioned 35 cm away. The UV-Vis illuminator (HSX-UV300, Beijing Newbit Technology Co., Ltd., Beijing, China) was operated at 15 A, with a spectral range of 200–2500 nm (covering UV, visible, near-infrared regions). Following 10 min UV irradiation, EPR analysis was conducted to determine •OH scavenging activity of CGA at different concentrations. EPR signal intensity of DMPO-OH• adducts was measured based on the amplitude of the second characteristic peak, calculated as peak height minus trough depth. The scavenging rate against •OH radicals is calculated according to the following formula, as specified in Supplementary Material Text S3.

SR = (I_0_ − I)/I_0_ × 100% (2)

where I_0_ denotes the amplitude value of the second characteristic peak in the EPR spectrum for the blank group, and I represents the corresponding value for the sample groups.

Time-dependence of CGA-mediated •OH scavenging activity: A 50 μL aqueous mixture containing 1 mM DMPO, 5 mM H_2_O_2_ and 0.35 mM CGA was studied. Post-UV irradiation, glass capillaries were placed into standard EPR quartz tubes for analysis; EPR measurements were conducted at 1–30 min irradiation time points to evaluate time-dependent •OH scavenging activity.

### 2.3. Fluorescence Imaging of Mouse Tissue In Vitro

Animals and fluorescein administration: A total of 8-week-old ICR mice (~25 g B.W) were purchased from GemPharmatech Co., Ltd. (Nanjing, China). After one week of acclimatization, the mice were randomly divided into two groups (n = 3 per group). The mice were intravenously injected via the tail vein with a single dose of 1 mg/kg FL or 2.4 mg/kg FL-CGA, respectively. All animals were sacrificed by cervical dislocation 10 min after injection.

Tissue Collection and Fixation: Major organs including heart, liver, spleen, lung, and kidney were rapidly harvested and rinsed with cold PBS (pH 7.4) to remove residual blood and contaminants. Tissues were immediately fixed in 4% paraformaldehyde (PFA) at 4 °C for 24 h to maintain morphology and preserve fluorescence signals.

Dehydration, paraffin embedding and sectioning: Fixed tissues were dehydrated through a graded ethanol series (70%, 80%, 90%, 95%, and 100% ethanol), cleared with xylene, and embedded in paraffin. Paraffin sections (5 μm) were prepared using a rotary microtome, mounted on positively charged slides, and dried overnight at 37 °C.

Deparaffinization and rehydration: Paraffin sections were sequentially treated with environment-friendly deparaffinization solution (10 min × 3) and anhydrous ethanol (5 min × 3), followed by rinsing with distilled water.

DAPI nuclear counterstaining: After gently removing excess liquid, the sections were incubated with DAPI staining solution in a light-tight humid chamber at room temperature for 10 min. The slides were then washed three times (5 min each) in pH 7.4 PBS with gentle agitation on an orbital shaker.

Slide mounting and fluorescence imaging: The slides were washed three times (5 min each) in PBS with gentle agitation on an orbital shaker. After removing excess liquid, the sections were mounted with anti-fade mounting medium. Fluorescence images were captured using a digital slide scanner (NanoZoomer S60, Hamamatsu Photonics, Hamamatsu, Japan) and analyzed with CaseViewer 2.6 software.

### 2.4. Cell Culture and Subculture Protocols

Mouse podocyte clone-5 (MPC-5, obtained from the Cell Bank of the Chinese Academy of Sciences Type Culture Collection) were used in this study. MPC-5 cells were seeded in RPMI-1640 medium supplemented with 5% fetal bovine serum (FBS) and 1% antibiotics, and cultured in a cell incubator at 37 °C with 5% CO_2_. When the cells reached approximately 90% confluence observed under a light microscope, subculture was performed. In a super-clean bench, the medium in the cell culture flask was aspirated, followed by rinsing twice with 1 mL of phosphate-buffered saline (PBS). After thorough aspiration of PBS, 2 mL of 0.25% trypsin-EDTA was added for digestion at 37 °C for 2 min, and then 2 mL of complete medium was added to terminate the digestion. The adherent cells at the bottom of the flask were gently pipetted to detach completely, and the trypsin cell suspension was transferred into a 15 mL centrifuge tube for centrifugation at 1000 rpm for 3 min. The supernatant was discarded, and the cell pellet was resuspended in 5 mL of complete medium (serum-containing medium). An appropriate volume of the cell suspension was seeded into a new culture flask pre-loaded with complete medium, mixed gently by shaking, and then placed back into the incubator for continuous culture. The cells were used for subsequent experiments after 2 passages.

### 2.5. Confocal Laser Scanning Microscopy Analysis

MPC-5 Cells were seeded in 35-mm transparent-bottom culture dishes (purchased from Shanghai Titan Scientific Co., Ltd., Shanghai, China). The cells were then treated with 0.22 μM FL or FL-CGA, respectively, and incubated for 12 h. After incubation, the cells were washed three times with PBS. The nucleus, endoplasmic reticulum, mitochondria and lysosomes were stained separately following the protocols of the corresponding commercial kits. After fixation with universal tissue fixative, the cells were observed under a Zeiss LSM 710 confocal laser scanning microscope (CLSM, Zeiss, Oberkochen, Germany).

## 3. Results

### 3.1. Antioxidant Activity of CGA Evaluated by EPR Spectroscopy

#### 3.1.1. Antioxidant Activity of CGA Against DPPH Radicals

DPPH radicals are characterized by a single-electron structure, a unique property that renders their typical spectral signals amenable to straightforward detection through electron EPR spectroscopy. As elaborated in our previous research [27], a well-defined linear relationship has been confirmed: the dynamic fluctuations in DPPH radical concentrations can be quantitatively delineated by the double integral of the characteristic peak in EPR spectra, a parameter that accordingly functions as a valid quantitative index for gauging the DPPH radical scavenging potency.

**Figure 1:**
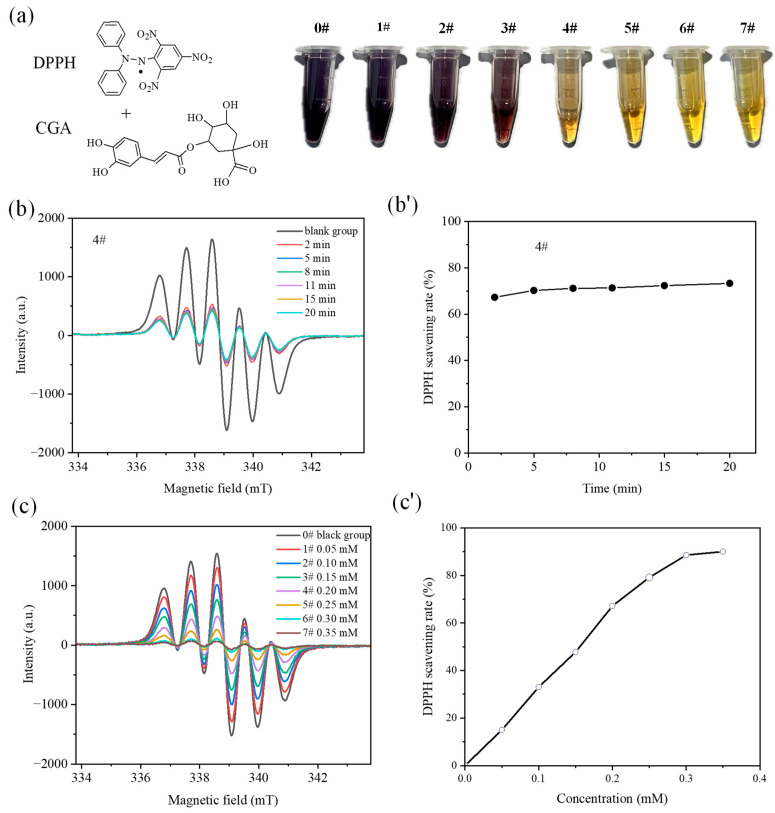
(**a**) Visual appearance of 0.5 mM DPPH solutions following 15 min incubation with chlorogenic CGA at gradient concentrations (Samples 0#–7#: 0.0, 0.05, 0.10, 0.15, 0.20, 0.25, 0.30, and 0.35 mM, respectively). (**b**,**b’**) EPR spectra and free radical scavenging rates of 0.5 mM DPPH solutions containing 0.20 mM CGA (Sample 4#) at various reaction time points. (**c**,**c’**) EPR spectra and corresponding free radical scavenging rates of 0.5 mM DPPH solutions treated with varying CGA concentrations for a 15-min incubation period.

Figure 1b,b’ depicts the time-dependent EPR spectra of 0.5 mM DPPH solutions treated with 0.2 mM CGA, along with the associated kinetic profile of DPPH radical scavenging activity over time. As shown in the figure, the reaction of CGA with DPPH proceeded with remarkable rapidity. The SR achieved 67.3% merely 2 min after the reaction commenced. Prolonging the reaction duration resulted in a progressive increase in SR, attaining 71.1% at approximately 8 min. Extending the reaction time further to 20 min induced only a marginal rise in SR to 73.2%, demonstrating that the maximum scavenging capacity of 0.2 mM CGA toward 0.5 mM DPPH radicals is constrained to this range.

Subsequently, EPR spectra and SR were determined following a 15 min incubation of different concentrations of CGA with the 0.5 mM DPPH solution, with results summarized in Figure 1c,c’. The data show that 0.05 mM CGA achieved a DPPH scavenging rate of 14.9%. With increasing concentrations of CGA, the SR displayed a linear increase, attaining a relatively high level of 88.5% when the concentration reached 0.30 mM; a further rise in CGA concentration caused the SR to level off, remaining steady at around 90.0%. Furthermore, the visual color changes of the solution (Figure 1a) serve as intuitive evidence for the radical-scavenging capacity of CGA. The blank DPPH solution exhibited a characteristic purple hue, whereas the solution color faded conspicuously following the addition of CGA at relatively high concentrations. After 20 min reaction, the solution turned pale yellow, particularly when the CGA concentration exceeded 0.25 mM. In contrast, the DPPH solution treated with CGA at concentrations lower than 0.15 mM remained distinctly purple even after the reaction time was prolonged to 60 min, which is a direct indication of a high residual level of unquenched DPPH radicals.

#### 3.1.2. Antioxidant Activity of CGA Against •OH

**Figure 2:**
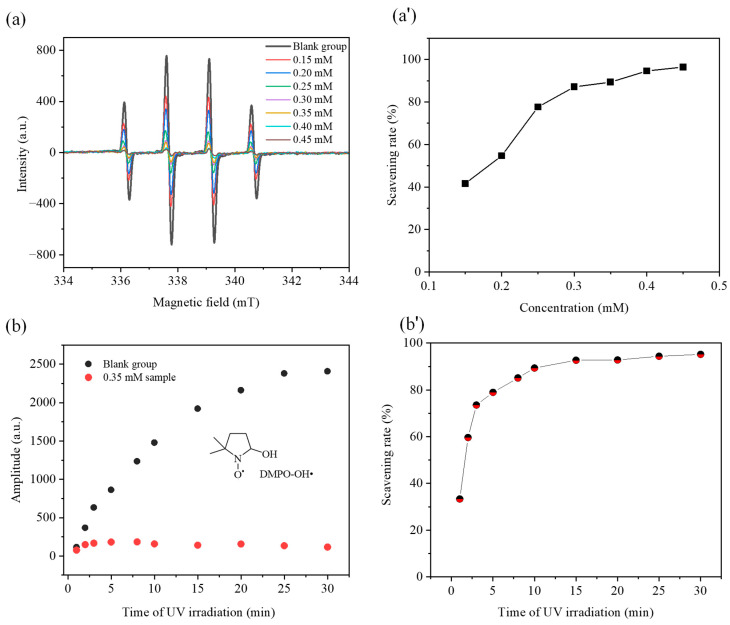
(**a**,**a’**) Dependence of EPR signal intensity and free radical scavenging rates of DMPO-OH• adducts (prepared with 5 mM H_2_O_2_ and 1 mM DMPO) on CGA concentrations ranging from 0.15 to 0.45 mM after 10 min of UV irradiation. (**b**,**b’**) Variations in EPR signal amplitude and scavenging rates of DMPO-OH• adducts (produced by the reaction system of 5 mM H_2_O_2_ + 1 mM DMPO) as a function of irradiation time, with a comparison between samples supplemented with 0.35 mM CGA and CGA-free blank controls.

•OH is a highly reactive free radical species that cannot be directly identified through EPR spectroscopy, primarily due to its extremely short half-life. To enable reliable detection of •OH, DMPO is widely used as a spin trap to form the relatively stable paramagnetic adduct DMPO-OH•. A xenon lamp was used as UV light source to irradiate a mixed solution of H_2_O_2_ and DMPO. Under UV irradiation, H_2_O_2_ underwent photodecomposition to generate •OH radicals, which were rapidly trapped by DMPO to form the stable DMPO-OH• adduct. As shown in Figure 2, the EPR signal intensity and SR of DMPO-OH• radicals were determined for the mixed systems containing 5 mM H_2_O_2_, 1 mM DMPO and CGA at gradient concentrations (0.1–0.45 mM) after 10 min of continuous UV irradiation. The results demonstrated that the peak height of the second characteristic signal in the EPR spectra was reduced with the introduction of CGA into the reaction system. Meanwhile, the •OH radical SR rose sharply with the increase in CGA concentration in the system: specifically, the SR of CGA reached 41.6% at a concentration of 0.15 mM and further climbed to 87.0% when the concentration was increased to 0.30 mM. In addition, the rate of increase in the •OH scavenging rate gradually slowed down with the continuous elevation of CGA concentration, and the maximum SR of 96.4% was finally achieved at the CGA concentration of 0.45 mM.

The •OH radical SR of the samples exposed to different UV irradiation durations is compared in Figure 2b,b’. The blank group (5 mM H_2_O_2_ + 1 mM DMPO) and the experimental sample group (5 mM H_2_O_2_ + 1 mM DMPO + 0.35 mM CGA) were both treated with UV irradiation of varying durations, following which the EPR spectra of the DMPO-OH• adduct were measured and recorded in real time. As shown in Figure 2b, the peak height of the second characteristic signal in the EPR spectra indicated that the EPR signal intensity of the DMPO-OH• adduct in the blank group increased in a linear manner with the extension of UV irradiation time. In sharp contrast, the corresponding signal intensity in the CGA-containing sample group remained at a consistently low level and showed almost no obvious change with the increase in irradiation duration. This result suggested that the added CGA could efficiently scavenge the •OH radicals generated by UV-induced photodecomposition. Figure 2b’ illustrates the •OH radical SR of the samples at different UV irradiation time points. In detail, the SR of the sample reached 33.3% after 1 min of UV irradiation and rose to 89.3% at the 10 min time point. With the further prolongation of UV exposure, the •OH radical SR increased moderately and continuously, reaching 92.8% and 95.2% at 20 min and 30 min of irradiation, respectively.

### 3.2. Fluorescence Distribution of FL and FL-CGA in Murine Tissues and Major Organs

**Figure 3:**
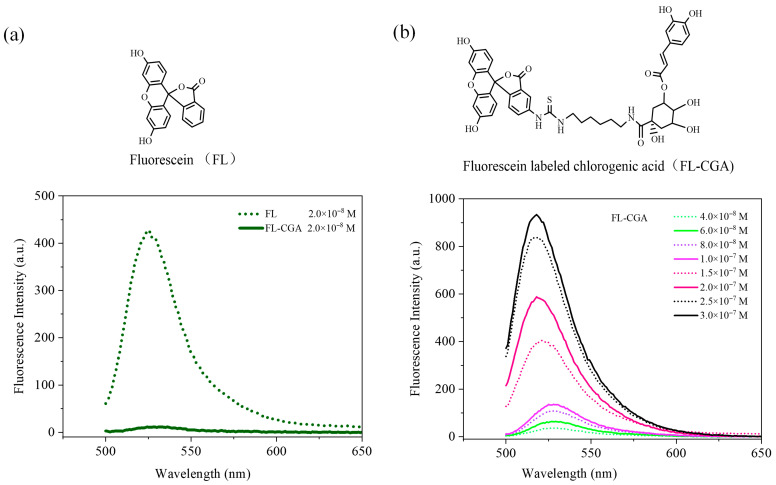
(**a**) Fluorescence intensity of FI and FL-CGA aqueous solution (2 × 10^−8^ M, excitation wavelength λ_ex_ = 491 nm); (**b**) Fluorescence intensity of FL-CGA aqueous solutions with different concentrations (4.0 × 10^−8^–3.0 × 10^−7^ M).

In this study, FL was employed to monitor the distribution of CGA in the major organs of mice. First, the fluorescence intensities of FL and FL-CGA were measured, as shown in Figure 3a. At the same concentration (2 × 10^−8^ M), the fluorescence intensity of FL was approximately 38 times that of FL-CGA. The results of fluorescence intensity measurements for FL-CGA at different concentrations are also presented in Figure 3b. The concentration of FL-CGA that yielded a fluorescence intensity comparable to that of FL was 1.5 × 10^−7^ M, which was 7.5 times the concentration of FL. These results provide a basis for the subsequent comparison of FL and FL-CGA concentrations via the relative fluorescence intensities of the probes in mice.

**Figure 4:**
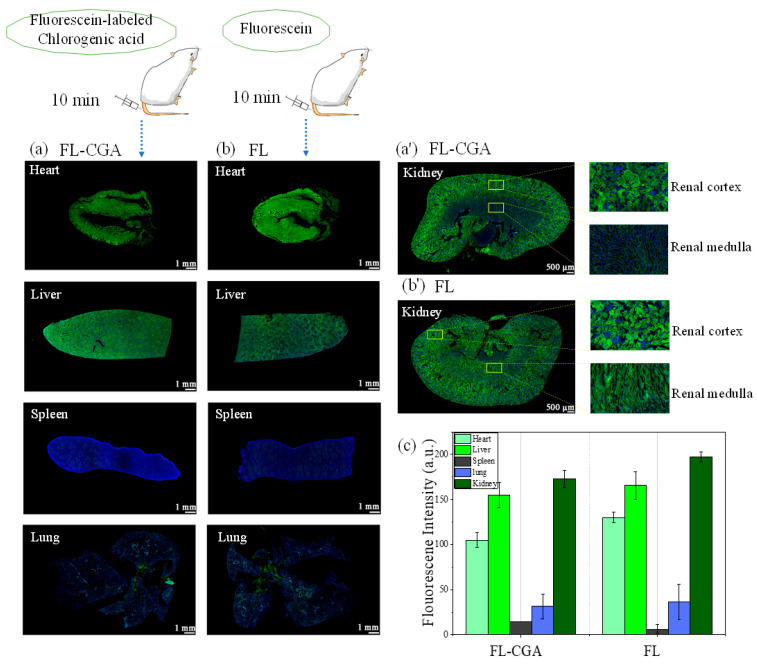
In Vivo fluorescence distribution of FL-CGA and FL (λ_ex_ = 490 nm, λ_em_ = 530 nm) in mouse tissues and major organs (heart, liver, spleen, lung, kidney): (**a**,**a’**) organs from mice injected with FL-CGA (1 mM, 3 mL/kg); (**b**,**b’**) organs from mice injected with FL (1 mM, 3 mL/kg); (**c**) Normalized fluorescence intensity of FL-CGA and FL in major organs.

Then, FL and FL-CGA samples were injected into the mice via the tail vein. After 10 min, the major organs were dissected and analyzed to monitor the distribution of CGA. As illustrated in Figure 4, fluorescence intensity analysis demonstrated that both FL and FL-CGA exhibited a similar distribution pattern across the major organs: low levels of fluorescence were detected in the spleen and lung, while relatively high fluorescence signals were observed in the kidney, liver and heart, with the kidney exhibiting the strongest fluorescence intensity. Further observations revealed intense fluorescence in both the renal cortex and medulla, suggesting that the fluorescently labeled CGA (FL-CGA) was successfully taken up by renal tubular and glomerular cells, thereby contributing its therapeutic efficacy. Compared with the FL-treated group, mouse kidney tissues administered with FL-CGA exhibited slightly weaker fluorescence intensity, which was approximately 88% of that measured in the FL group. Based on the above comparison between the fluorescence intensity and concentration of FL and FL-CGA, the fluorescence intensity of FL-CGA in aqueous solution at the same concentration was only 8% that of FL. This result indicates that the conjugate of CGA and fluorescein is more readily taken up by the kidney than free fluorescein alone. In addition, the phenolic hydroxyl groups of CGA are the major functional groups responsible for its antioxidant activity. Therefore, we conjugated FL to the carboxyl group of CGA to maximally preserve its biological activity. A supplementary in vitro experiment compared the radical scavenging capacities of 0.2 mM CGA, FL, and FL-CGA against 0.5 mM DPPH using EPR spectroscopy (Figure S1 in the Supplementary Material). The results revealed that the DPPH scavenging rates of CGA, FL, and FL-CGA were 72.3%, 5.97%, and 80.9%, respectively, indicating that the phenolic antioxidant activity of CGA was well retained in FL-CGA.

**Figure 5:**
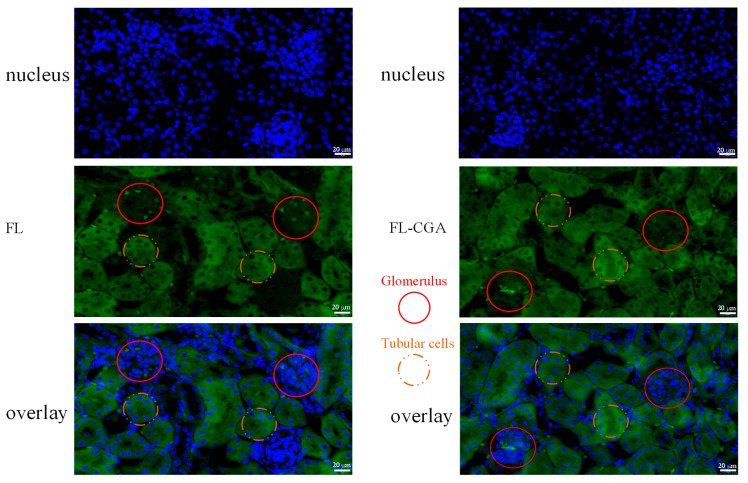
Representative high-magnification images showing the distribution and localization of FL-CGA and FL in mouse kidney.

Figure 5 shows representative high-magnification images illustrating the localization of FL-CGA and FL in the mouse kidney. Red solid circles denote the glomerulus, the core filtration unit of the kidney, while orange dashed circles denote tubular cells. Overlay images clearly demonstrate that FL and FL-CGA signals are widely distributed across both glomerular and tubular regions.

### 3.3. The Confocal Fluorescence Microscopy Imaging of FL and FL-CGA in Podocytes

**Figure 6:**
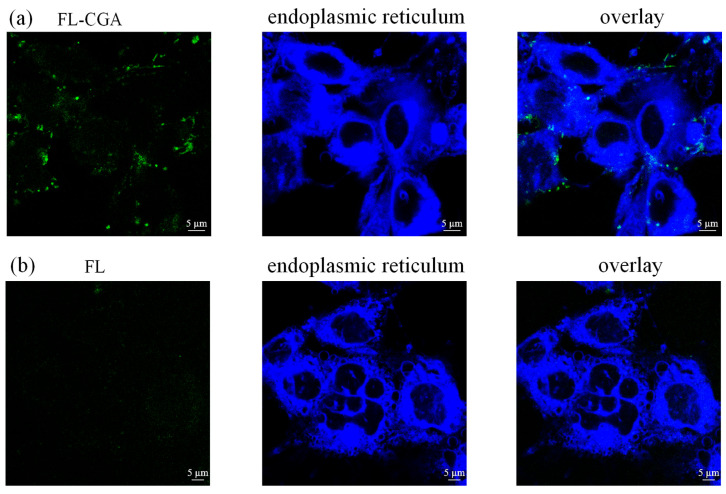
Confocal fluorescence microscopy images of MPC-5 cells following incubation with FL-CGA or FL (22 μM) after 12 h. (**a**) FL-CGA signal (λ_ex_ = 488 nm, λ_em_ = 563 nm), endoplasmic reticulum staining (λ_ex_ = 405 nm, λ_em_ = 451 nm), and the corresponding merged overlay; (**b**) FL signal (λ_ex_ = 488 nm, λ_em_ = 563 nm), endoplasmic reticulum staining, and the corresponding merged overlay.

**Figure 7:**
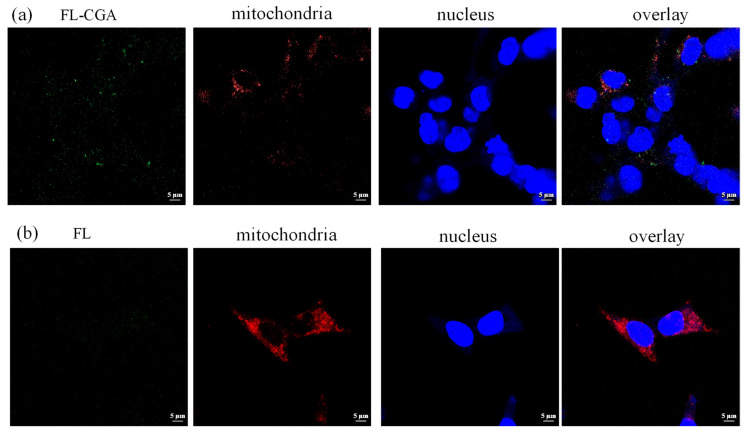
Confocal fluorescence microscopy images of MPC-5 cells following incubation with FL-CGA or FL (22 μM) after 12 h. (**a**) FL-CGA signal, mitochondrion staining (λ_ex_ = 543 nm, λ_em_ = 615 nm), nucleus staining (λ_ex_ = 405 nm, λ_em_ = 451 nm), and the corresponding merged overlay; (**b**) FL signal, mitochondrion staining, nuclear staining, and the corresponding merged overlay.

**Figure 8:**
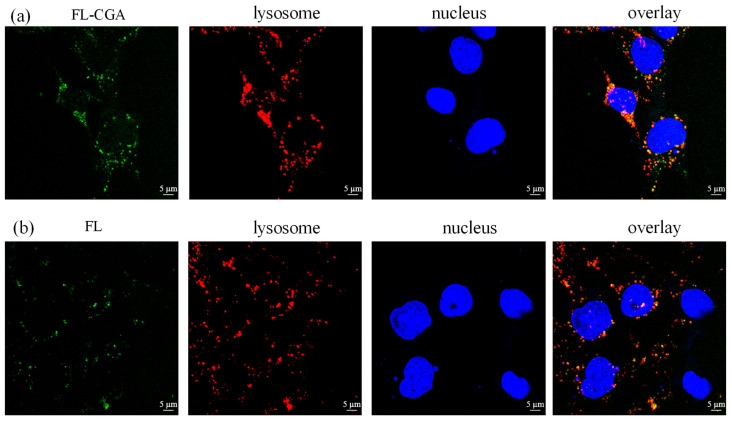
Confocal fluorescence microscopy images of glomerular cells following incubation with FL-CGA or FL (22 μM) after 12 h. (**a**) FL-CGA signal, lysosome staining (λ_ex_ = 543 nm, λ_em_ = 615 nm), nucleus staining (λ_ex_ = 405 nm, λ_em_ = 451 nm), and the corresponding merged overlay; (**b**) FL signal, lysosomal staining, nuclear staining, and the corresponding merged overlay.

Fluorescent imaging of MPC-5 cells incubated with FL or FL-CGA was conducted using a confocal fluorescence microscope. As presented in Figure 6, Figure 7 and Figure 8, only the fluorescence of stained lysosomes overlapped with that of FL-CGA. The results demonstrated that both FL-CGA and FL could specifically enter the lysosomes of MPC-5 cells but did not localize to the endoplasmic reticulum or mitochondria, and this enriching property may lay a foundation for the therapeutic effects of CGA. Furthermore, under identical incubation conditions with equimolar concentrations of the fluorescent probes, the lysosomal fluorescence intensity generated by FL-CGA was significantly higher than that generated by FL alone. This difference is attributable to the more efficient internalization of CGA by MPC-5 cells. Lysosomes, often referred to as the “intracellular digestive workshop”, are catabolic organelles crucial for the degradation of intracellular and extracellular components through processes like autophagy, endocytosis, phagocytosis, and micropinocytosis. Lysosomal dysfunction is widely recognized as a critical factor in various cellular pathologies, inflammatory responses, and disease progression [28,29,30]. Concurrently, abnormal activation of endoplasmic reticulum and mitochondria frequently initiates cellular stress or apoptosis. Endoplasmic reticulum is vital for protein synthesis, folding, and lipid metabolism, and its disruption leads to endoplasmic reticulum stress [31]. Mitochondria are also central to cellular homeostasis, with their dysfunction contributing to disease states through mechanisms like ATP depletion, ROS production, and the initiation of apoptosis [32]. By precise lysosomal accumulation, FL-CGA may avoid interfering with core physiological processes such as endoplasmic reticulum protein folding and mitochondrial energy metabolism, thereby reducing cytotoxicity caused by off-target effects and improving therapeutic safety.

## 4. Discussion

Cellular oxidative stress is prevalent in various chronic kidney diseases and serves as a core mechanism driving disease progression [33]. Effective alleviation of oxidative stress using natural products is of great significance for the prevention and treatment of various nephropathies [4,5]. The antioxidant capacity of plant extracts is routinely evaluated by determining their free radical scavenging activity. This study employed EPR spectroscopy to investigate the antioxidant activity of CGA in vitro, particularly its ability to scavenge •OH radicals. Furthermore, a fluorescence tracing technique using fluorescently labeled CGA provided direct visual evidence for the spatial distribution of FL-CGA in mouse organs and its uptake by renal podocytes. The strategy established in this study, which integrates in vivo fluorescence tracing of CGA in mice with in vitro antioxidant evaluations, also provides a methodological insight for the intervention studies of dietary polyphenols in CKD.

In contrast to traditional spectrophotometric methods including UV–visible spectroscopy for analyzing the antioxidant activity of CGA [15,16,17], EPR spectroscopy allows direct and specific detection of paramagnetic substances [34], thus avoiding common interference caused by endogenous colored compounds in plant extracts [35,36,37]. In the present work, we characterized the antioxidant activity of CGA by monitoring EPR signal variations of free radicals (DPPH and •OH) before and after their scavenging by CGA. The CGA concentrations used in the EPR assay (0.2–0.45 mM, equivalent to 0.071–0.159 mg/mL) were chosen to ensure effective scavenging of 0.5 mM DPPH radicals while meeting the EPR detection limit for quantification. According to published data [38], one serving of unblended roasted and ground coffee contains 194.1 ± 67.7 mg CGA, which is consistent with the concentration range applied herein. DPPH, a stable nitrogen-centered free radical, was chosen as a representative probe owing to its widespread application in evaluating in vitro antioxidant capacity. Despite the high stability of DPPH radicals, CGA displayed exceptionally rapid DPPH-scavenging activity. Following the addition of 0.2 mM CGA to 0.5 mM DPPH solution, the characteristic EPR signal intensity of DPPH reached a steady state within only 2 min of incubation, clearly indicating the potent and rapid free radical-quenching behavior of CGA. Notably, the DPPH-scavenging efficiency of CGA determined by EPR spectroscopy was consistent with previously reported IC50 values derived from conventional spectrophotometric assays [15].

We further evaluated the scavenging capacity of CGA toward •OH radicals, short-lived and physiologically relevant oxidants that are closely associated with oxidative stress injury. Given its extremely short half-life, •OH was trapped by DMPO to form the stable paramagnetic spin adduct DMPO-OH•, and the inhibitory effect on DMPO-OH• signal intensity was used to assess the •OH-scavenging ability of CGA. Unlike previous studies that employed hydroxyl radical generation systems based on the Fenton reaction and peroxydisulfate [19,39], the present study utilized a light-induced H_2_O_2_ system to generate hydroxyl radicals in real time. This approach offers the advantage of dynamically regulating the concentration of hydroxyl radicals by adjusting the irradiation time [27]. The results demonstrated that 0.45 mM CGA remarkably suppressed the formation of DMPO-OH• adducts generated via UV irradiation in a system containing 5 mM H_2_O_2_ and 1 mM DMPO, with an inhibition ratio of 96.4% after 10 min of UV irradiation.

In addition, FL-CGA was predominantly distributed in the kidneys following intravenous injection via the mouse tail vein. Mouse kidney tissues administered with FL-CGA exhibited slightly lower fluorescence intensity than those with FL. However, for FL-CGA to achieve a comparable fluorescence intensity to FL, it required 7.5 times the concentration of FL, yet the overall renal uptake of FL-CGA appeared more efficient than free fluorescein alone. This suggests that while the fluorophore itself might contribute to some uptake, the CGA moiety likely facilitates more specific interactions or transport mechanisms, leading to enhanced kidney accumulation compared to free fluorescein. In addition, fluorescence localization revealed that FL-CGA accumulated in the renal tubules, with relatively weak signals in the glomeruli. Although some renoprotective agents preferentially accumulate in renal tubular cells, they can also exert protective effects against primary (e.g., membranous nephropathy) and secondary (e.g., diabetic kidney disease) glomerular injuries. Podocytes are highly specialized, characteristic cells of the glomerulus and a critical component of the glomerular filtration barrier [40]. We therefore investigated the subcellular localization of FL-CGA in podocytes to clarify its renoprotective mechanisms.

Cellular assays further revealed that FL-CGA was taken up by podocytes and mainly localized within lysosomes, providing a novel perspective for elucidating the renoprotective mechanism of CGA. Such lysosome-targeted distribution may be partially attributed to fluorescein conjugation. Given the molecular size and physicochemical properties of FL-CGA, endocytosis represents a highly plausible uptake pathway. Although this mechanism has not been directly demonstrated for CGA, certain polyphenols and their conjugates bind specific cell-surface receptors, triggering receptor-mediated endocytosis and subsequent lysosomal delivery, analogous to the reported behavior of epigallocatechin gallate (EGCG) conjugated with bovine serum albumin (BSA) [41]. It has been documented that BSA-based conjugates undergo endocytosis and are selectively transported into lysosomes. These findings are also consistent with previous studies demonstrating that natural polyphenols and flavonoids, including resveratrol, quercetin, and EGCG, can scavenge ROS and preserve or restore lysosomal function. Specifically, resveratrol has been reported to attenuate pulmonary fibrosis, enhance autophagic flux, and regulate the autophagy–lysosome pathway, possibly by promoting the formation of autolysosomes [42]. Quercetin protects pancreatic β-cells against palmitic acid-induced apoptosis by restoring lysosomal function and autophagic flux, thus improving cell viability [43]. Meanwhile, EGCG preserves lysosomal membrane stability via its antioxidant and antihypercholesterolemic activities by neutralizing free radicals, thereby alleviating lipid accumulation in aged lysosomes [44].

Our study primarily focuses on the intrinsic antioxidant activity and tissue/cellular targeting of the CGA molecule itself (or its fluorescein-labeled derivative). Although it does not directly demonstrate the advantages of dietary CGA, it provides foundational evidence for the potential therapeutic efficacy of this naturally occurring compound. Owing to the complex interplay between dietary components and gut microbiota [10], the overall health benefits of CGA-rich foods are likely synergistic, involving both parent CGA and its diverse metabolites [45].

Collectively, combining assays based on stable radicals and physiologically relevant transient radicals enables a more comprehensive evaluation of the antioxidant activity of CGA. Owing to limitations including cellular permeability, metabolic degradation, and the intricate redox microenvironment in vivo, results from DPPH and •OH scavenging assays may not be directly extrapolated to complex biological systems. Preliminary cellular experiments confirmed that CGA protects MPC-5 cells against H_2_O_2_-induced viability impairment (Text S4 in the Supplementary Material). The CCK-8 assay showed that different treatments significantly altered cell viability. Compared with the control group, the 0.5 mM H_2_O_2_ group exhibited markedly reduced cell viability, decreasing to 11.8% of the control level (*p* < 0.05). In contrast, following co-treatment with 0.5 mM H_2_O_2_ and 50 μM CGA, cell viability recovered to 33.2% of the control level (*p* < 0.05).

Future studies will adopt clinically relevant podocyte injury models induced by high glucose and oxidative stress. CGA or FL-CGA pretreatment will be applied to evaluate cell viability, apoptosis, inflammation, and barrier function. Measurements of intracellular ROS in stressed podocytes will validate the cellular antioxidant activity of CGA, linking its in vitro radical-scavenging capacity to cytoprotection. In vivo, clinically relevant CKD models (e.g., STZ-induced nephropathy and 5/6 nephrectomy) combined with FL-CGA tracing will be employed, and renal histopathology, renal function, and oxidative stress markers will be examined to correlate the antioxidant activity of CGA with renoprotection.

## 5. Conclusions

This study systematically evaluated the antioxidant activity of CGA using EPR spectroscopy, confirming its potent scavenging capacity against both stable DPPH radicals and highly reactive, cytotoxic •OH radicals. Using a fluorescein-labeled CGA derivative (FL-CGA) as a molecular probe, we visualized its biodistribution in major murine organs and characterized its subcellular localization in podocytes. FL-CGA exhibited prominent accumulation in both the renal cortex and medulla. Following internalization by podocytes, it specifically localized to lysosomes, with negligible signal detected in the endoplasmic reticulum or mitochondria. Collectively, these findings establish a critical link between CGA’s robust antioxidant capacity and its targeted accumulation in renal lysosomes, providing novel experimental evidence to support CGA as a potential dietary intervention for CKD via the attenuation of oxidative stress. While this study remains preliminary for direct clinical and translation applications, it lays a solid foundation for future comprehensive investigations into CGA’s renoprotective mechanisms.

## Data Availability

The original contributions presented in this study are included in the article. Further inquiries can be directed to the corresponding author.

## Supplementary Materials

The following supporting information can be downloaded at: https://www.mdpi.com/article/10.3390/nu18081181/s1. Text S1: The methods of EPR measurements; Text S2: DPPH radicals scavenging rate calculation formula; Text S3: •OH radicals scavenging rate calculation formula; Text S4: CGA protects MPC-5 cells against H_2_O_2_-induced viability impairment; Figure S1: Radical scavenging capacities of CGA, FL, and FL-CGA against DPPH (EPR spectroscopy, in vitro).

## Abbreviations

The following abbreviations are used in this manuscript:

CGA	Chlorogenic acid
FL-CGA	Fluorescein-labeled chlorogenic acid
CKD	Chronic kidney disease
EPR	Electron paramagnetic resonance
ROS	Reactive oxygen species
DPPH	1,1-diphenyl-2-picrylhydrazyl
DMPO	5,5-dimethyl-1-pyrroline *N*-oxide
•OH	Hydroxyl radical
DMPO-OH•	DMPO and •OH adduct
STZ	Streptozotocin
DN	Diabetic nephropathy
Nrf2	Nuclear factor E2-related factor 2
ABTS	2,2′-azino-bis(3-ethylbenzothiazoline-6-sulphonic acid)
SR	Scavenging rate
FBS	Fetal bovine serum
MPC-5	Mouse podocyte clone-5
PBS	Phosphate-buffered saline
DAPI	4′,6-diamidino-2-phenylindole
