# Supplementary material for: Antioxidant Activity of Chlorogenic Acid Evaluated via EPR Spectroscopy and Its Visual Tracking in Mouse Kidney
Source: Nutrients. 2026 Apr 9;18(8):1181. doi: 10.3390/nu18081181 (PMC13118533; doi:10.3390/nu18081181)
Supplement: Supplementary file 1 [file nutrients-18-01181-s001.zip › nutrients-4200899-supplementary.pdf]

Supplementary Materials

Antioxidant Activity of Chlorogenic Acid Evaluated via EPR Spectroscopy and Its Visual Tracking in Mouse Kidney

Li Quan [1,2], Cheng Li [3], Peipei Shen [3], Enchao Zhou [1,2,*], Gui Yin[3] and Xuewen Guo [3,*]

[1] The First Clinical Medical College, Nanjing University of Chinese Medicine, Nanjing 210023, China
[2] Jiangsu University Key Laboratory of Tonifying Kidney and Anti-senescence, Nanjing 210023, China
[3] School of Chemistry, Centre for Shared Scientific Research Facilities, Nanjing University, Nanjing 210023, China
[*] Correspondence: zhouenchao@njucm.edu.cn; guoxuewen@nju.edu.cn

Text S1

The electron paramagnetic resonance (EPR) spectroscopy used was Magnettech ESR5000 Benchtop EPR Spectrometer (Bruker BioSpin GmbH, Ettlingen, Germany). EPR measurement was carried out in a glass capillary tube (inner diameter: 0.9-1.0 mm) containing approximately 50 μL of the sample solution sealed with rubber putty and placed into a standard EPR quartz tube (inner diameter: 3 mm). The tube was then placed in the resonator cavity of the EPR spectrometer. The settings used for EPR spectroscopy were as follows: Central magnetic field, 338.40 mT; Sweep width: 15 mT; Microwave power: 10 mW; Modulation frequency: 100 kHz; Modulation amplitude: 0.2 mT; Sweep time, 60 s; Test temperature: 25 ºC.

Text S2

Time-dependence of DPPH radical scavenging activity by CGA: Using ethanol as the solvent, DPPH was subjected to serial dilution to prepare a standard solution with a concentration of 5 mM. Meanwhile, CGA was dissolved in deionized water to obtain a stock solution at a concentration of 2 mM. Subsequently, 100 μL of the CGA stock solution was mixed thoroughly with an equal volume (100 μL) of the 5 mM DPPH solution. The resulting mixture was then adjusted to a final volume of 1000 μL with ethanol, yielding a reaction system containing 0.5 mM DPPH and 0.2 mM CGA. EPR spectra of this reaction system were periodically recorded at different time intervals.

The area under the characteristic DPPH peaks was calculated via double integration of the corrected EPR spectra using Bruker ESR Studio software (Version 1.90.1, Bruker BioSpin GmbH).

The DPPH radical scavenging rate (SR) is expressed by the following formula:

$$SR = (A_0 - A) / A_0 \times 100\% \cdots\cdots \text{(Eq. S1)}$$

where A_0 denotes the double integral area under the characteristic peaks of the EPR spectrum for the blank group, and A represents that of the EPR spectra for the sample groups.

Dependence of DPPH radical scavenging activity on CGA concentration: Aliquots (100 μL) of CGA solutions with initial concentrations ranging from 0.5 to 3.5 mM (deionized water as the blank control) were mixed with an equal volume (100 μL) of 5 mM DPPH solution, and the mixture was adjusted to a final volume of 1000 μL with ethanol. The reaction system was incubated in the dark for 15 min, followed by EPR measurement.

Text S3

Dependence of •OH scavenging activity on CGA concentration: Equal volumes (90 μL each) of 10 mM DMPO, 50 mM H_2O_2 and CGA solutions at concentrations ranging from 1.5 to 4.5 mM were mixed thoroughly. The resulting mixture was diluted to a final volume of 900 μL using deionized water. Subsequently, 50 μL aliquots of the mixed solution were transferred into glass capillary tubes, which were then sealed with rubber putty. The sealed capillaries were irradiated using a xenon lamp positioned 35 cm away from the samples. The illumination was provided by a UV-Vis light source (model HSX-UV300, Beijing Newbit Technology Co., Ltd.) operating at a current of 15 A. The spectral output ranged from 200 nm to 2500 nm, encompassing the ultraviolet, visible, and near-infrared regions. The mixture was exposed to UV irradiation for 10 min, followed by EPR spectroscopic analysis to determine the •OH radical scavenging activity of CGA at different concentration gradients. The EPR signal intensity of DMPO-OH• adducts was quantified based on the amplitude value of the second characteristic peak in the spectra, defined as the difference between the peak height and trough depth.

The •OH scavenging rate (SR) is calculated according to the following formula:

$$SR = (I_0 - I) / I_0 \times 100\% \cdots\cdots \text{(Eq. S2)}$$

where I_0 denotes the amplitude value of the second characteristic peak in the EPR spectrum for the blank group, and I represents the corresponding value for the sample

groups.

Time-dependence of •OH radical scavenging activity by CGA: Equal volumes (90 μL each) of 10 mM DMPO, 50 mM H_2O_2 and 3.5 mM CGA solution (deionized water was used as the blank control) were mixed and diluted to a final volume of 900 μL with deionized water. After UV irradiation, the glass capillary tubes were placed into standard EPR quartz tubes for EPR spectroscopic analysis. EPR measurements were performed at irradiation time points ranging from 1 to 30 min to assess the time-dependent radical scavenging activity.

Text S4

Preliminary experiment on the effect of CGA against H_2O_2-induced injury in MPC-5 cell viability

The MPC-5 cell line was used in this study. Cells were seeded in 96-well plates and cultured until they reached approximately 70% confluency. Subsequently, the cells were divided into four groups: control group, a 0.5 mM H_2O_2 group, and groups treated with 0.5 mM H_2O_2 combined with either 25 μM or 50 μM CGA, respectively. After 5 hours of treatment, the culture medium was aspirated, and CCK-8 reagent (Beijing Labgic Technology Co., Ltd.) was added to each well, followed by a 2-h incubation. Absorbance was measured using a CLARIOstar microplate reader (BMG LABTECH GmbH, Ortenberg, Germany), and cell viability was calculated accordingly.

The CCK-8 assay showed that different treatments significantly altered cell viability. Compared with the control group, the 0.5 mM H_2O_2 group exhibited markedly reduced cell viability, decreasing to 11.8% of the control level ($P < 0.05$). In contrast, CGA could ameliorate H_2O_2-induced cell damage. Specifically, upon co-treatment with 0.5 mM H_2O_2 and 25 μM or 50 μM CGA, cell viability recovered to 20.4% ($P < 0.05$) and 33.2% ($P < 0.05$) of the control, respectively. These findings indicate that, within the tested concentration range, CGA improves the viability of H_2O_2-injured MPC-5 cells in a dose-dependent manner.

Figure S1

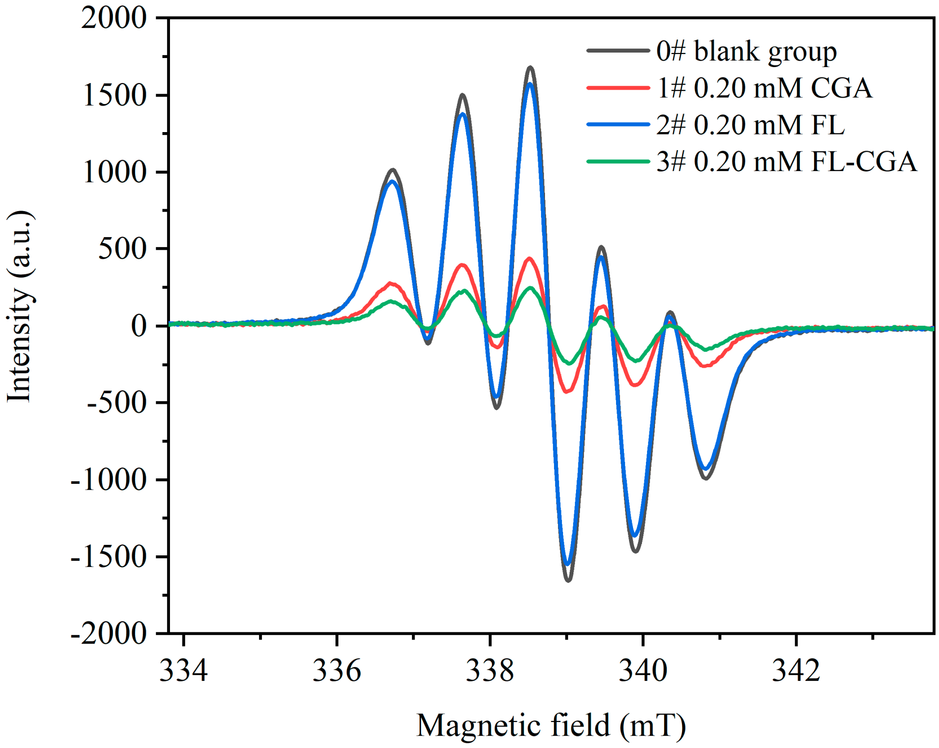

Figure S1. EPR spectra of 0.5 mM DPPH solution, as well as DPPH solutions treated with 0.20 mM CGA, FL, and FL-CGA after 15 minutes of incubation.
